# Atypical Modulations of N170 Component during Emotional Processing and Their Links to Social Behaviors in Ex-combatants

**DOI:** 10.3389/fnhum.2017.00244

**Published:** 2017-05-23

**Authors:** Sandra P. Trujillo, Stella Valencia, Natalia Trujillo, Juan E. Ugarriza, Mónica V. Rodríguez, Jorge Rendón, David A. Pineda, José D. López, Agustín Ibañez, Mario A. Parra

**Affiliations:** ^1^Doctoral Program in Psychology, Department of Experimental Psychology, Universidad de GranadaGranada, Spain; ^2^GISAME, Facultad Nacional de Salud Pública, Universidad de Antioquia (UdeA),Medellín, Colombia; ^3^Neuroscience Group, Universidad de Antioquia (UdeA),Medellín, Colombia; ^4^Facultad de Jurisprudencia, Universidad del RosarioBogotá, Colombia; ^5^SISTEMIC, Facultad de Ingeniería, Universidad de Antioquia (UdeA),Medellín, Colombia; ^6^Neuropsychology and Behavior Group, Universidad de Antioquia (UdeA),Medellín, Colombia; ^7^Department of Cognitive Neuroscience, Maastricht UniversityMaastricht, Netherlands; ^8^Facultad de Psicología, Universidad Autónoma del CaribeBarranquilla, Colombia; ^9^Center for Social and Cognitive Neuroscience (CSCN), School of Psychology, Universidad Adolfo IbañezSantiago, Chile; ^10^National Scientific and Technical Research CouncilBuenos Aires, Argentina; ^11^Laboratory of Experimental Psychology and Neuroscience, Institute of Translational and Cognitive Neuroscience, INECO Foundation, Favaloro UniversityBuenos Aires, Argentina; ^12^ACR Centre of Excellence in Cognition and its Disorders, SydneyNSW, Australia; ^13^Psychology, School of Social Sciences, Heriot-Watt UniversityEdinburgh, United Kingdom

**Keywords:** emotional processing, N170, social neurosciences, social behavior, ex-combatants

## Abstract

Emotional processing (EP) is crucial for the elaboration and implementation of adaptive social strategies. EP is also necessary for the expression of social cognition and behavior (SCB) patterns. It is well-known that war contexts induce socio-emotional atypical functioning, in particular for those who participate in combats. Thus, ex-combatants represent an ideal non-clinical population to explore EP modulation and to evaluate its relation with SCB. The aim of this study was to explore EP and its relation with SCB dimensions such as empathy, theory of mind and social skills in a sample of 50 subjects, of which 30 were ex-combatants from illegally armed groups in Colombia, and 20 controls without combat experience. We adapted an Emotional Recognition Task for faces and words and synchronized it with electroencephalographic recording. Ex-combatants presented with higher assertion skills and showed more pronounced brain responses to faces than Controls. They did not show the bias toward anger observed in control participants whereby the latter group was more likely to misclassify neutral faces as angry. However, ex-combatants showed an atypical word valence processing. That is, words with different emotions yielded no differences in N170 modulations. SCB variables were successfully predicted by neurocognitive variables. Our results suggest that in ex-combatants the links between EP and SCB functions are reorganized. This may reflect neurocognitive modulations associated to chronic exposure to war experiences.

## Introduction

Emotional processing (EP) relies on fast neural mechanisms which are crucial for promoting adaptive survival strategies (Plutchik, 2001; Adolphs, 2003; Brown et al., 2010; Barratt and Bundesen, 2012; LoBue and Rakison, 2013). Facial expressions allow individuals to quickly identify other people’s emotional status (Ekman and Oster, 1979; Batty and Taylor, 2003; Eimer and Holmes, 2007; Luo et al., 2010; Recio et al., 2011; Weymar et al., 2011; Sawada et al., 2014). Similarly, a fast categorization has also been observed when emotional content is conveyed by words (Kanske and Kotz, 2007; Ibáñez et al., 2011; Rohr and Rahman, 2015). The early identification of emotional information provides clues which are necessary for social interactions, e.g., to anticipate potential threats.

Recent studies have shown that early neural markers of EP can predict critical dimensions of social cognition and behavior (SCB) (Hurtado et al., 2009; Ibáñez et al., 2014; Kawamoto et al., 2014; Dozolme et al., 2015; Zinchenko et al., 2015). For instance, Petroni et al. (2011) reported in healthy university students associations of the electrophysiological marker N170, sensitive to emotional valence, and SCB dimensions informing on theory of mind (ToM) and executive functions. Ibáñez et al. (2014) reported atypical event related potentials (ERPs) modulations during the analysis of the Stimulus Type Effect (i.e., greater amplitudes during face relative to word processing over the right hemisphere) in patients with schizophrenia. These patients show a reduced cortical activation over the right hemisphere during face processing in comparison with words. Although, it should be noted that such a differential increase in N170 amplitude seen in patients with schizophrenia might be contingent upon the contrasted stimuli as others have found the opposite pattern when faces are contrasted to buildings rather than words (Herrmann et al., 2004). Ibáñez et al. (2014) also suggested that modulations of the N170 component during EP of faces are a sensitive predictor of social and cognitive performance in healthy participants as well as in patients with schizophrenia, attentional-deficit/hyperactivity disorder and bipolar disorders. Taken together, such evidence suggests an association between early cortical markers of EP and SCB. It follows that subjects with aberrant behavioral and neural markers of EP would present with impaired SCB (Hall et al., 2004).

In support to this proposal, it has been found that impairments during the processing of faces conveying emotional expressions are associated with atypical social responses in individuals with brain damage (Adolphs et al., 2002), autism (Adolphs et al., 2001), schizophrenia (Hooker and Park, 2002), and psychopathy (Decety et al., 2014). An important population to investigate this hypothesis is that of ex-combatants. They represent a non-clinical group with well-documented impairments in EP (Tobón et al., 2015; Quintero-Zea et al., 2017; Trujillo et al., 2017). Ex-combatants typically display diminished empathic expressions (McCarroll et al., 2010; Slep et al., 2010), increased levels of aggression and violence (Jakupcak et al., 2007; Taft et al., 2007; Gallaway et al., 2012), high proportion of a wide variety of mental disorders (Taft et al., 2007; Weierstall et al., 2013; Kaplan and Nussio, 2015, 2016) as well as high emotional reactivity reflected via emotion related ERP components (IAPS; Lang et al., 1988; Tobón et al., 2015).

Chronic exposure to war experiences may lead to a gradual implementation of adaptive mechanisms, which may render EP in these individual atypical as compared to controls. Our main hypothesis is that ex-combatants’ SCB have undergone reorganization due to their exposure to war experiences and such reorganization could be accounted for by associations between atypical modulations of emotion related ERP components (i.e., N170; Ibáñez et al., 2014) and EP responses, such as poorer behavioral performance during emotional recognition tasks (ERTs). Moreover, we hypothesized that ERP markers of EP would be associated with SCB in ex-combatants and control groups, and would account for lower EP performance in ex-combatants.

The decision to focus on the N170 component was entirely theory-driven. Although the role of N170 as a pure physiological measure of EP remains controversial, evidence has accrued suggesting that in the context of emotional recognition paradigms as the one used in our study, it does index the neural responses to the emotional valence of faces and words (Schacht and Sommer, 2009; Petroni et al., 2011; Ibáñez et al., 2014). N170 provides a sensitive marker of EP during both semantic (Luo et al., 2010; Ibáñez et al., 2011, 2014; Zhang et al., 2014; Chen et al., 2015) and facial processing (Batty and Taylor, 2003; Luo et al., 2010; Meaux et al., 2014). There is evidence that EP forms part of the repository of functions supporting SCB components such as empathy, ToM, and social skills (Petroni et al., 2011; Melloni et al., 2014; Quintero-Zea et al., 2017; Trujillo et al., 2017). Social cognition is known to rely on perceptual integration which subserves the interpretation of human interactions (Andreou et al., 2015) such as inference of emotional stages, intentions, beliefs and reasoning of others (Adolphs, 2001; Petroni et al., 2011; Stanley and Adolphs, 2013; Kalin et al., 2015; Bora et al., 2016). For the purpose of the present study, and in line with previous reports (Quintero-Zea et al., 2017; Trujillo et al., 2017), we decided to include three measures of social cognition indexing ToM and empathy [i.e., The Interpersonal Reactivity Index (IRI) (Davis, 1983); the Read the Mind in the Eyes (Baron-Cohen et al., 2001); and the Hinting task (Gil et al., 2012)]. We also included measures of social behaviors which are sensitive to explore ecological patterns of interactions during daily social contacts. We included self-report measures (Gismero, 2000) which incorporate multidimensional constructs of social skills. In this study, we interpret SCB as a construct derived from the estimation of cognitive resources used during social interactions and the evaluation of explicit social responses. Thus, we considered SCB a multidimensional domain assessed with measures of ToM, empathy and social interactions. This framework was used to investigate the extent to which modulations of the N170 component during a word and face emotional categorization task would serve as a marker of EP and if so, whether they would predict SCB patterns in Colombian ex-combatants and controls.

## Materials and Methods

### Participants

The sample consisted of 54 participants. Of these, 34 were ex-combatants from illegal groups of the Colombian armed conflict who, by the time of the study, were enrolled in the reintegration program offered by “Agencia Colombiana para la Reintegración”^1^. A trained psychologist (NT) performed an initial short individual interview who collected the history of psychiatric and neurological disorders that had required medical care. Those requiring such care were excluded from the study. We also excluded individuals that were not able to perform the task. The decision about excluding participants was made by the research group after careful consideration of the outcomes from the interview and before the electroencephalography (EEG) session. We excluded four ex-combatants due to substance dependence (2), history of cerebrovascular disease (1) and active pharmacological treatment for depression (1). The final sample consisted of 20 Controls and 30 Ex-combatants. Both groups were matched according to age, gender, and years of education (**Table 1**). Ex-combatants were mainly men (28 men and 28 were right-handed), their ages ranged from 27 to 57, and had an average education of 10.23 years (*SD* = 3.03). The control group consisted of 20 volunteers (18 men, 19 right handed) with ages ranging between 24 and 55 years and a mean education of 11.05 years (*SD* = 2.14). All the participants read and signed the informed consent before starting the study. The study procedures and informed consent was approved by Ethics Committee of the Faculty of Medicine Universidad de Antioquia, Medellín, Colombia. Participants were informed about the aim of the study, the confidentiality of the information collected and also about procedures of psychological tests and electroencephalographic recordings. Only when the participants signed the consent form the professionals in charge started the evaluation.

**Table 1:** Mean data and statistical analysis for group comparisons using demographic and social cognition dimension variables from ex-combatants and controls.

	Ex-combatants (*n* = 30) M *(SD)*	Controls (*n* = 20) M *(SD)*	*t*/*Chi^2^* (P)
Demographic			
Age	37.50 (8.22)	36.1 (9.17)	0.54 (0.59)
Gender (F:M)	2:28	2:18	0.41 (0.52)
Years of education	10.23 (3.03)	11.05 (2.14)	-1.12 (0.27)
Laterality (L:R)	3:27	1:19	0.18 (0.67)
Social cognition			
IRI PT	16.90 (4.91)	15.60 (4.08)	1.00 (0.34)
IRI FS	13.30 (4.68)	13.25 (5.10)	0.03 (0.97)
IRI EC	19.13 (4.66)	18.45 (2.82)	0.59 (0.56)
IRI PD	11.23 (5.10)	13.50 (4.78)	-1.58 (0.12)
Hinting task	17.77 (2.20)	16.68 (4.78)	0.93 (0.29)
RMIE	19.76 (5.80)	19.89 (4.24)	-0.08 (0.94)
GSSS	68.10 (15.86)	80.40 (25.56)	-***2.10 (0.04)***
SS1	15.60 (5.67)	18.00 (7.94)	-1.25 (0.22)
SS2	10.43 (3.07)	10.60 (4.61)	-0.15 (0.88)
SS3	10.33 (2.89)	11.55 (3.85)	-1.27 (0.21)
SS4	11.47 (4.12)	16.20 (5.67)	-***3.42 (0.00)***
SS5	9.30 (3.45)	10.85 (4.07)	-1.45 (0.15)
SS6	10.97 (3.44)	13.20 (4.07)	-***2.9 (0.04)***

*IRI PT, perspective taken; IRI FS, fantasy; IRI EC, empathic concern; IRI PD, personal distress; RMIE, Reading the mind in the eyes; GSSS, Global social skills score; SS1, auto-expression in social situations; SS2, defense of the rights as consumer; SS3, anger or unconformity expression; SS4, to say not and to cut interactions; SS5, make petitions; SS6, initiate interactions with opposed sex. Bold and italic represent significant effects.*

### Assessment of Cognition and Social Behavior

#### Theory of Mind

We used the revised version of the Reading the Mind in the Eyes task (Baron-Cohen et al., 2001). The task consists of 36 photos of the eyes area (males and females) portraying different emotional expressions. Each image was surrounded by five possible answers, of which only one was correct and the others were distractors. Participants had to indicate, by selecting the appropriate option, the emotion they saw in the image of the eyes.

The Hinting task, created by Corcoran et al. (1995) evaluated the inference of social intentions. It consists of 10 short stories that represent the interaction between two characters. At the end of each story, one of the characters provides an obvious hint. Subjects were asked to evaluate what the hint really meant within the story’s context.

#### Empathy

The Spanish version of the empathy scale IRI of Davis (1983) was used (Escrivá et al., 2004). This scale evaluates dispositional cognitive and emotional empathic dimensions. The scale has 28 self-report items of which 19 are presented in a positive frame, and the remaining 9 in a negative one. Responses are entered using a five-point Likert scale (1 = it does not describe me well to 5 = it describes me very well). The scale is divided into four dimensions: Perspective Taking (PT), Empathic Concern (EC), Fantasy (FS), and Personal Distress (PD). PT evaluates the ability to consider other’s points of view. EC assesses the response to feelings of compassion or sympathy through recognizing others misfortunes. FS explores the ability to self-identify as a fictional character in a story such as novels, books or movies. PD measures self-oriented negative arousal in response to stressors, attitudes, and experiences of other people.

#### Measures of Social Skills

The social ability scale of Gismero (2000) is a self-report instrument that evaluates everyday social behaviors via 33 items. This scale inquiries individuals about their ability to interact with others in different situations. Items are grouped in six dimensions: (1) self-expression in social situations, (2) defense of own rights as a consumer, (3) expression of anger or displeasure, (4) stop interactions and saying no, (5) make requests, (6) start positive interactions with the opposite gender. Responses are recorded using a four-point Likert scale (1 = I do not identify with that at all/most of the time it does not happen/I would not do it to 4 = I totally agree/most of the time/I would behave like that). The scale has an alpha Cronbach of 0.88 and has demonstrated to be sensitive to social skills variations in normal populations (Gismero, 2000). Larger values of this score suggest reduced social assertion.

#### Emotion Recognition Task (ERT)

A task for identifying faces and words with emotional content was implemented in E-prime (Psychology Software Tools, Pittsburgh, PA, United States). The stimuli consisted of 90 pictures of female and male faces (30 happy, 30 neutral, and 30 angry) which were taken from the MMI Facial Expression Database (Pantic et al., 2005). Additionally, 90 words (30 pleasant, 30 neutral, and 30 unpleasant) were selected from the linguistic corpus generated by the communications department at University of Antioquia (Preseea, 2005). The Linguistic corpus contains the list of words more frequently used in Antioquia, Colombia (Preseea, 2005). From the corpus we selected words with two to three syllabi with the highest frequency in the metropolitan area of the city, categorized as positive, neutral or negative in content according to the report by Preseea (2005). Both faces and words were adapted following Ibáñez et al. (2011) methodology. The stimuli were presented on a 17′′ PC screen placed 60 cm away from the participants’ eyes. In addition, a pilot study was carried out to corroborate the validity of the adapted task to investigate emotional recognition discrimination. We found a large overall precision (around 90%), differential reactions times across conditions and the Stimulus Type effect reported by Rossion et al. (2003) (See details of the pilot study in Supplementary Material 1)

**FIGURE 1:**
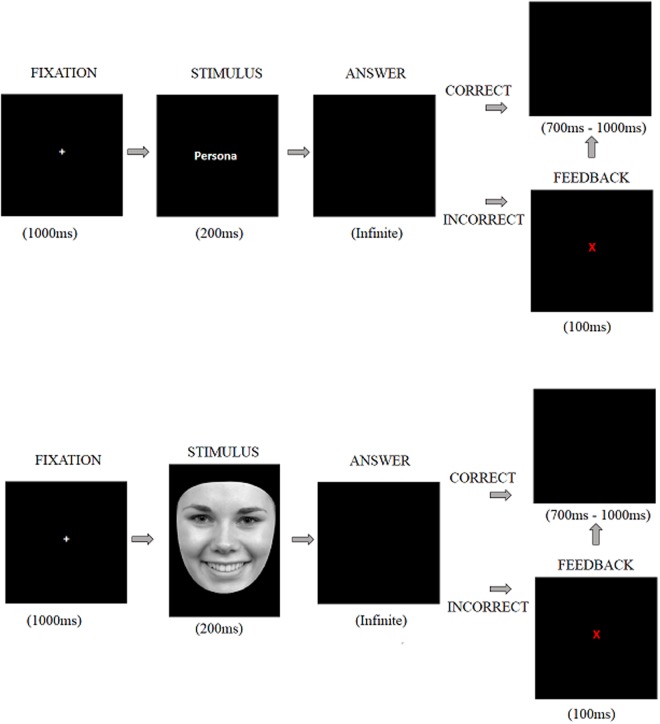
**An example trial for each stimulus category of the Emotion Recognition Task**.

The task sequence is shown in **Figure 1**. A fixation cross was presented for 1000 ms which was followed by the stimulus display (i.e., face or word) presented for 200 ms. immediately after, the participant’s response was requested. If the stimulus was a face, they were asked to decide whether it showed a happy, neutral, or angry expression. If the stimulus was a word, they were asked to decide whether it described a pleasant, neutral, or unpleasant emotion. Participants entered their responses by pressing one of three keys previously allocated of a standard PC keyboard. Correct responses were followed by a black screen which appeared for a random duration between 700 and 1000 ms (i.e., intertrial interval). The incorrect response was indicated by a red letter “X” which appeared in the center of the screen for 100 ms. this feedback was used to encourage attention to the task. The feedback screen was followed by the inter-trial interval described above.

The experiment was divided into two blocks, each with 180 stimuli. Each block consisted of 45 faces and 45 words (15 happy, 15 neutral and 15 angry faces, and 15 pleasant, 15 neutral and 15 unpleasant words). Each stimulus was presented twice in a random order with faces and words intermixed in the same sequence always avoiding more than two consecutive stimuli of the same valence. We calculated reaction time, accuracy and types of errors (i.e., the proportion of response erroneously assigned from the other two conditions).

#### EEG Recordings

The ERT was synchronized with EEG recordings. EEG signals were acquired with a 64-channel EEG NeuroScan SynAmps2 sampling at a frequency of 1 KHz. Quick-caps were placed according to the 10–20 system. Impedances were kept below 10 kΩ. Recording sessions were carried out in a Faraday cage of 2x1 m with dimmed light.

#### Signal Processing

Electroencephalography recordings were pre-processed in EEGLab toolbox ran on Matlab 2012 (Delorme and Makeig, 2004). The original signals were downsampled from 1000 to 500 Hz and offline re-referenced to mastoids. In order to reduce the environmental artifacts, a band-pass Infinite Impulse Response (IIR) digital filter was applied (0.1–30 Hz). An Independent Component Analysis (ICA) for EEGlab (Delorme and Makeig, 2004) was performed in order to remove electrooculography (EOG) artifacts. A maximum of two artifact components were removed. Thereafter, signals were reconstructed to their original configuration.

Each Task (Face/Word) and Condition (Happy/Pleasant, Neutral/Neutral, and Angry/Unpleasant) was epoched with 1s windows (-200 to 800 ms). Epochs were baseline corrected using the window -200 to 0 ms. Additionally, each epoched signal was visually inspected to manually remove the remaining artifacts.

### Procedures

The application of the SCB scales and the ERT was counterbalanced across participants whereby half of the sample received the SCB scales first and the ERT second, and the other half received them in the opposite order.

### Statistical Analysis

Demographic variables and SCB scales were analyzed using independent-samples *t*-test or Chi2 (for gender) (see **Table 1**). To analyze the behavioral data, two two-way mixed ANOVA models were used, one for Faces and one for Words. Condition (Happy/Pleasant vs. Neutral/Neutral vs. Angry/Unpleasant) was the within-subjects factor and Group (Ex-combatants vs. Controls) the between-subjects factor. We entered reaction time and accuracy (percentage of correct responses). For the sake of brevity, we report in the manuscript Group by Condition interactions (for the main effects see Supplementary Material 2). In addition, Errors were calculated as the proportion of responses within each alternative valence (i.e., Type) erroneously assigned to the judged valence. For example, if the stimulus presented a Neutral face, two types of errors could be committed, i.e., Neutral-Happy whereby the subject identifies a happy emotion or Neutral-Angry whereby the subject identifies an angry emotion. The analysis of Error Type across task conditions is relevant as it would inform whether poor accuracy is driven by a particular bias toward a specific emotion and whether the pattern of bias differs across groups. The same two-way ANOVA model was used to analyze the Type of Error (Type of Error 1 vs. Type of Error 2) and Group (G1 vs. G2).

To analyze the ERP (N170) data, we implemented a four-way repeated-measures ANOVA for amplitude and latency, in which Task (Face vs. Word), Condition [Positive (Happy/Pleasant) vs. Neutral (Neutral Face/Neutral Word) vs. Negative (Angry/Unpleasant)], and Hemisphere (Left vs. Right) were the within-subjects factors and Group (Ex-combatants vs. Controls) was the between-subjects factor. In the model, we considered that the relation between Task and Hemisphere would be crucial to investigate the presence of the Stimulus Type Effect described by Rossion et al. (2003) and Ibáñez et al. (2014). For the sake of brevity, we focus on the significant interactions that involved Group. To further explore significant interactions, we used Bonferroni corrected *post hoc* tests adjusting the Alfa level according to the number contrasts. For the interactions we calculated effect size (η^2^: 0.1 = small, 0.24 = medium, and 0.31 large) and power (β), whereas for *post hoc* analyses the effect size was calculated using the Cohen’s *d* (0.2 = small, 0.5 = medium, and 0.8 = large).

To explore whether SCB could be predicted by neurocognitive functions (i.e., behavioral: accuracy/reaction time and electrophysiological: amplitude/latency) which proved informative of between-group differences, we ran a stepwise multiple regression analysis. Following the aim of the present study, SCB dimensions were the dependent variables and neurocognitive functions (i.e., EP behavioral and ERP variables) were the predictors.

## Results

### Social Cognition and Behavior

No between-group differences were observed for age, education, ToM or empathy. Ex-combatants showed higher social skills than Controls in domains such as “saying no and cutting interactions,” and “initiating interactions with the opposite gender.” Hence, ex-combatants showed higher assertion skills than Controls. **Table 1** shows demographic data and SCB scores from the two groups.

### ERT: Behavioral Data

For the Face Task neither accuracy [*F*(2,45) = 0.91, *p* = 0.39, η^2^ = 0.14, β = 0.19] nor reaction time [*F*(2,45) = 1.01, *p* = 0.37, η^2^ = 0.21, β = 0.22] yielded significant Group × Condition interactions. The same outcomes were observed for the Word Task [Accuracy: *F*(2,45) = 0.43, *p* = 0.6, η^2^ = 0.09, β = 0.11; Reaction Time: *F*(2,45) = 0.02, *p* = 0.98, η^2^ = 0.03, β = 0.05].

For the Face Task, the Type Error during the Neutral Condition yielded a significant interaction [*F*(1,47) = 4.56, *p* = 0.04, η^2^ = 0.30, β = 0.55]. *Post hoc* analyses carried our across the Types of Error revealed that Controls committed more errors of the type Neutral answered erroneously Angry compared to the type Neutral answered erroneously Happy [*t* = 3.2, *p* = 0.01, *d* = 0.73]. No difference were found for ex-combatants across the Type of Errors in the Neutral Condition [*t* = 0.03, *p* = 0.97, *d* = 0.01]. No interaction with Group were observed for the Types of Error during Happy [*F*(1,47) = 0.88, *p* = 0.35, η^2^ = 0.13, β = 0.15] or Angry faces [*F*(1,47) = 0.38, *p* = 0.54, η^2^ = 0.09, β = 0.09]. In sum, Controls but not ex-combatants were more biased toward angry than happy emotions when they saw neutral faces.

For the Word Task no significant Type of Error × Group interaction was found for Pleasant [*F*(1,47) = 1.03, *p* = 0.32, η^2^ = 0.14, β = 0.17], Neutral [*F*(1,47) = 0.51, *p* = 0.48, η^2^ = 0.10, β = 0.11], or Unpleasant words [*F*(1,47) = 0.73, *p* = 0.40, η^2^ = 0.12, β = 0.13]. **Table 2** shows the mean and standard deviation for accuracy, type of error, and reaction time for each condition of the Face and Word tasks.

**Table 2:** Mean and standard deviation for Reaction Time, Accuracy and Type of Error for Faces and Words Conditions in emotional recognition task (ERT).

	Ex-combatants M (*SD*)	Controls M (*SD*)
**Faces**		
**Reaction time (ms)**		
Happy	908.68 (316.97)	1190.87 (599.95)
Neutral	980.52 (268.94)	1357.31 (579.79)
Angry	983.51 (267.90)	1242.03 (560.44)
**Accuracy (%)**		
Happy	82.81 (19.12)	87.02 (18.70)
Neutral	61.55 (24.09)	64.31 (22.53)
Angry	62.93 (21.76)	73.68 (18.90)
**Errors (%)**		
Happy	17.18 (19.13)	12.98 (18.70)
Happy error neutral^∗^	6.32 (8.74)	6.84 (13.35)
Happy error angry	10.86 (13.76)	6.14 (7.63)
Neutral	38.45 (24.09)	35.69 (22.53)
Neutral error happy	19.05 (16.49)	13.04 (12.76)
Neutral error angry	19.40 (12.71)	22.65 (13.29)
Angry	37.01 (21.78)	24.74 (14.84)
Angry error happy	11.49 (14.90)	6.93 (11.30)
Angry error neutral	25.51 (17.28)	17.80 (11.30)
**Words**		
**Reaction time (ms)**		
Pleasant	1032.67 (301.63)	1242.51 (487.77)
Neutral	1111.98 (416.91)	1329.25 (449.28)
Unpleasant	1115.75 (350.90)	1338.17 (456.43)
**Accuracy (%)**		
Pleasant	69.02 (20.24)	81.89 (13.45)
Neutral	56.12 (21.81)	64.61 (19.28)
Unpleasant	65.17 (25.16)	78.42 (17.85)
**Errors (%)**		
Pleasant	20.98 (20.80)	18.10 (13.45)
Pleasant error neutral	16.95 (12.12)	12.84 (9.76)
Pleasant error unpleasant	14.02 (14.77)	5.26 (7.47)
Neutral	43.92 (21.85)	35.39 (19.28)
Neutral error pleasant	30.89 (13.18)	28.54 (16.51)
Neutral error unpleasant	13.03 (13.17)	6.84 (7.63)
Unpleasant	34.83 (25.16)	21.58 (17.85)
Unpleasant error pleasant	17.87 (17.61)	8.94 (8.59)
Unpleasant error neutral	16.95 (15.82)	12.63 (11.86)

*^∗^On Error Type the first label (i.e., Happy) correspond to the expected respond whereas the second label represent the erroneous answer (i.e., Neutral) given by the subject.*

### Emotion Recognition Task: ERP Data

Mean amplitude and latency data are presented in **Table 3**. **Table 4** shows main effects and statistical interactions. Using amplitude data, we found a significant main effect of Task whereby Faces yielded greater amplitudes than Words (*MSE* = 0.62, *p* = 0.00, *CI* = 0.32–0.93). The main effect of Condition was also significant indicating that Positive stimuli (Happy/Pleasant) elicited larger N170 than Neutral (*MSE* = 0.12, *p* = 0.03, *CI* = 0.09–0.23) and Negative stimuli (Angry/Unpleasant) (*MSE* = 0.13, *p* = 0.04, *CI* = 0.04–0.26). N170 amplitude for Neutral and Negative stimuli did not differ (*MSE* = 0.08, *p* = 0.84, *CI* = -0.07 to 0.09). No other main effect was found to be significant.

**Table 3:** Mean amplitude and latency of N170 across Face and Word Conditions (Happy/Pleasant vs. Neutral/Neutral vs. Angry/Unpleasant) in Left and Right Hemisphere, in Ex-combatants and Controls.

	Ex-combatants M (*SD*)				Controls M (*SD*)			
	Amplitude (uV)		Latency (ms)		Amplitude (uV)		Latency (ms)	
	Left	Right	Left	Right	Left	Right	Left	Right
Happy	4.08 (1.63)	4.70 (2.25)	240.40 (14.12)	243.27 (12.01)	3.67 (2.16)	4.48 (1.99)	231.50 (20.71)	226.50 (29.13)
Neutral	4.09 (1.45)	4.69 (2.13)	239.93 (14.08)	243.40 (11.11)	3.80 (2.03)	4.59 (2.16)	230.40 (19.72)	236 (25.76)
Angry	3.98 (1.75)	4.57 (2.29)	239.53 (13.24)	241.67 (15.12)	3.80 (2.00)	4.49 (1.96)	231 (20.55)	236.60 (23.81)
Pleasant	3.33 (1.66)	3.52 (1.96)	227.73 (13.67)	221.07 (21.16)	3.77 (2.06)	4.59 (2.16)	224.9 (11.87)	236 (25.77)
Neutral	3.28 (1.56)	3.51 (1.80)	231.13 (14.37)	226.80 (18.71)	3.89 (2.29)	3.33 (1.56)	226 (12.58)	221.60 (15.87)
Unpleasant	3.20 (1.56)	3.50 (1.80)	228.33 (15.66)	223.47 (16.20)	4.03 (2.37)	3.54 (1.84)	226.90 (9.21)	218.70 (16.08)

**Table 4:** Results from the statistical analysis of the ERP data illustrating main effects and interactions.

	Amplitude	Latency
	*F*(p), η^2^, β	*F*(p), η^2^, β
Task	***16.63 (0.00), 0.51, 0.98***	***40.03 (0.00), 0.67, 1***
Condition	***3.69 (0.03), 0.26, 0.67***	0.74 (0.48), 0.12, 0.13
Hemisphere	2.70 (0.11), 0.22, 0.36	0.02 (0.88), 0.02, 0.05
Group	0.08 (0.78), 0.04, 0.06	2.33 (0.13), 0.21, 0.32
Task × Group	***4.98 (0.03), 0.30, 0.59***	***6.59 (0.01), 0.34, 0.71***
Condition × Group	2.0 (0.14), 0.37, 0.40	1.69 (0.19), 0.18, 0.35
Hemisphere × Group	0.28 (0.87), 0.03, 0.05	0.49 (0.49), 0.10, 0.11
Task × Condition	***6.00 (0.03), 0.33, 0.87***	1.65 (0.19), 0.18, 0.34
Task × Hemisphere	***7.98 (0.01), 0.37, 0.79***	3.65 (0.06), 0.27, 0.47
Condition × Hemisphere	***11.73 (0.00), 0.44, 0.99***	0.33 (0.72), 0.08, 0.10
Task × Condition × Group	***5.37 (0.01), 0.32, 0.83***	***5.48 (0.01), 0.32, 0.84***
Task × Hemisphere × Group	1.20 (0.28), 0.15, 0.19	0.98 (0.33), 0.14, 0.16
Task × Condition × Hemisphere	***8.35 (0.00), 0.38, 0.96***	***5.64 (0.01), 0.32, 0.85***
Condition × Hemisphere × Group	***13.88 (0.00), 0.47, 0.99***	0.56 (0.57), 0.11, 0.14
Task × Condition × Hemisphere × Group	***11.07 (0.00), 0.43, 0.99***	***7.71 (0.00), 0.37, 0.94***

*Bold and italic represent significant effects.*

**FIGURE 2:**
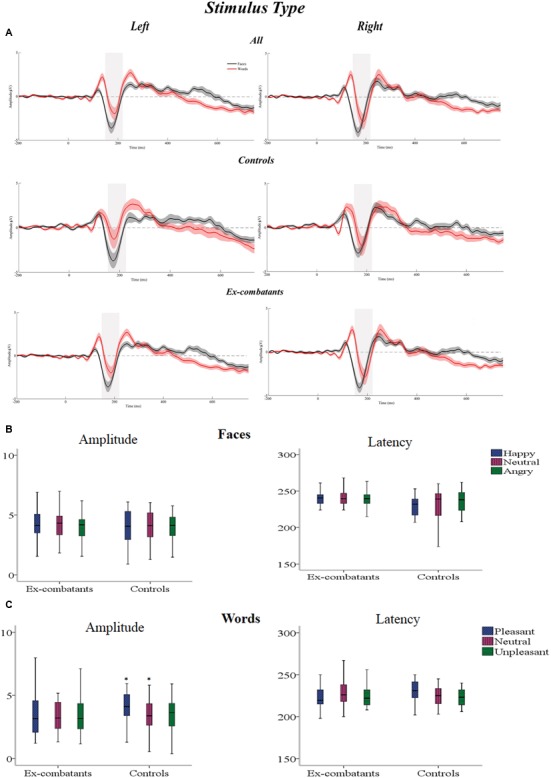
**(A)** Stimulus Type Effect: graphs show waveforms for Face and Word Task from the Right and Left Hemisphere from Controls and Ex-combatants respectively. **(B)** Mean amplitude and latency for the Face Task across the two groups. **(C)** Mean amplitude and latency for the Word Task across the two groups. ^∗^Significant differences.

The statistical interaction informing about the Stimulus-Type Effect (Task × Hemisphere) was significant. *Post hoc* contrasts were carried out across the two Factors [Task (2) × Hemisphere (2) = 4; adjusted-α = 0.01]. The factor Task showed that Faces elicited a larger N170 component over the Right than over Left Hemisphere [*t* = 2.67, *p* = 0.01, *d* = 0.36]. This differential activation was not present for Words. *Post hoc* contrasts carried out for each Hemisphere across Tasks showed that it was only over the Right Hemisphere that Faces elicited greater activation than words [*t* = 5.1, *p* < 0.001, *d* = 0.51]. Looking at other interactions, we found that the Task × Group interaction was also significant. *Post hoc* contrasts carried out across Tasks show that Faces elicited larger N170 component than Words, and effect observed in Ex-combatants only [*t*(29) = 4.75, *p* < 0.001, *d* = 0.60]. No other *post hoc* contrasts revealed significant effects (see **Figure 2**). In sum, the analysis of the Stimulus Type Effect not only confirmed the presence of this effect in the investigated groups but also revealed a significantly larger effect during face processing in Ex-combatants than in Controls.

The three-way Task × Condition × Group interaction was significant. *Post hoc* contrasts were carried out across the three Factors [Task (2) × Condition (3) × Group (2) = 12; adjusted-α = 0.004]. For the sake of comparability we explored *post hoc* contrasts for each Task separately. The Face Task revealed no significant differences across the contrasted factors. For the Word Task, Condition yielded a statistically significant difference only for Controls and for the contrast between Pleasant vs. Neutral stimuli [*t*(19) = 3.61, *p* < 0.001, *d* = 0.71]. No other *post hoc* contrasts revealed significant effects. There three-way Condition × Hemisphere × Group interaction was significant. However, *post hoc* contrasts carried out as described above failed to reach the significance threshold. Finally, although the four-way Task × Condition × Hemisphere × Group interaction was found to be significant, none of the performed *post hoc* contrasts reached the corrected threshold (2 × 3 × 2 × 2 = 24; adjusted-α = 0.002). In sum, the set of interactions found to be significant during the analysis of the N170 amplitude revealed that only Controls reacted to the emotional content of words and neither Controls nor Ex-combatants reacted to the emotional content of faces.

Taken together, these ERP findings suggest that Faces elicited greater activation than Words over the right hemisphere in both groups. However, Ex-combatants’ face reactivity was more pronounced than that seen in Controls. Processing emotions conveyed by Faces did not generate differential activations while emotions conveyed by words did but only in Controls.

The same model was used to analyze the Latency of the N170 component. **Table 4** shows main effects and statistical interactions. There was a significant effect of Task whereby shorter latencies characterized N170 for Words than for Faces than (*MSE* = -0.11, *p* = 0.00, *IC* = -0.11 to -0.70). Task interacted with Group. *Post hoc* contrasts (adjusted-α = 0.01) showed that Words elicited a faster N170 component in Ex-combatants [*t*(29) = 8.61, *p* = 0.00, *d* = 1.19] with no significant effects in Controls. Contrasts carried out for each Task separately and between groups revealed no significant effects. Other interactions which were found to be significant were those between Task × Condition × Group and Task × Condition × Hemisphere × Group. However, corrected *post hoc* tests carried out to explore theses interaction were non-significant. In sum, the set of interactions found to be significant during the analysis of the N170 latency revealed that Words are processed faster than Faces, an effect drove by the Ex-combatant Group.

Finally, the stepwise regression model incorporated Social Skills as the dependent variables [Global social skills score (GSSS); auto-expression in social situations (SS1); defense of the rights as consumer (SS2); anger or unconformity expression (SS3); to say not and to cut interactions (SS4); make petitions (SS5) and initiate interactions with opposed sex (SS6) dimensions independently], and N170 latency for Words, N170 amplitude for Faces, N170 amplitude for Neutral and Pleasant Words, and Type Error during Neutral Faces (i.e., error happy and error angry) as the predictors. The analysis revealed that the “auto-expression in social situations” was significantly predicted by Type of Error during Neutral Faces (error happy) [*B* = 0.33; *F*(1,48) = 5.66; *p* = 0.02]. Moreover, the “expression of anger and displeasure” was significantly predicted by N170 latency for Words [*B* = -0.34; *F*(1,48) = 5.96; *p* = 0.02]. These models explained 33% and 34% of the variance respectively. No other associations were found to be significant.

## Discussion

This study was set out to investigate the extent to which modulations of the N170 component elicited during an ERT that presents Words and Faces would serve as a marker of EP and whether they would predict SCB in Colombian ex-combatants and Controls. We found that (1) Ex-combatants presented with higher assertion skills than Controls. (2) The previously reported Stimulus Type Effect was present in both groups and Ex-combatant showed an exacerbated response to Faces (i.e., N170). (3) Ex-combatants were less likely than controls to misclassify Neutral Faces but showed an atypical word valence processing. (4) The efficiency to process neutral stimuli and the N170 latency for Words significantly predicted SCB functions. These results have important implications for our understanding of the bio-psycho-social consequences of war conflicts and their influence on social behaviors. We now discuss such implications.

The first three findings of our study suggest that chronic exposure to war experiences may reshape the EP system as to become more efficient for socially relevant cues. Ex-combatants showed higher scores than Controls in some areas of social assertion which inform about abilities to interact in heterogeneous environments. The demands posed by rapidly changing violent contexts such as those wherein Ex-combatants regularly interact may require continues readjustments of SCB skills. From this perspective, it is not entirely surprising to observe higher social assertion in Ex-combatants than in individuals who were not directly exposed to violent contexts. Ex-combatants also showed better abilities to cut interactions and to establish social relationships with members of the opposite gender. Similar features have been found in subjects with antisocial behaviors (Raine, 2002; Glenn et al., 2007). Our view is that members of armed groups are continuously trained to modulate their emotions and generate pragmatic social responses which allow them to successfully evaluate complex contexts and map social situations arising from these contexts to adaptive behaviors. Further research will be necessary to identify factors accounting for these adaptive mechanisms such as roles taken in war scenarios, length of the exposure to war conflicts, and SCB features prior to war conflicts. Moreover, future research will be needed to identify if such adaptive mechanisms are characteristic of ex-combatants in Colombia or in other societies.

A second finding suggest that some form of functional reorganization at a social level in Ex-combatants are linked to the Stimulus Type Effect (Rossion, 2014). Ex-combatants seem to rely on carriers of social cues which are more relevant to their environments. This study reveals that Faces appear to be more relevant to Ex-combatants than Words. Not only they showed the well-known Stimulus Type Effect as Controls did, but their neural responses to Faces was more pronounced overall than that of Controls. Behaviorally, Ex-Combatants were less likely to misclassify Neutral Faces. The Stimulus Type Effect has been considered a marker of the spatio-temporal properties of stimuli and their distribution in the visual network (Rossion et al., 2003). Our findings in Ex-combatants suggest that although they are unresponsive to the emotional valence of stimuli whether shown by faces or words, they process words quickly and faces more slowly and deeply, thus suggesting a superior value of visual information over verbal information for this group. Ex-combatants seem to have developed high-level visual processing skills which are necessary for a fast recognition of salient aspects of visual scenes, such as faces (Rossion, 2014). One might argue that this process, which is fundamental for successful social interactions (Ibáñez et al., 2014), may undergo reorganization following prolonged exposure to war experiences.

Words proved less informative in Ex-combatants than in Controls. Differential activation of words with emotional valence relative to neutral words has been previously reported (Kissler et al., 2006). The emotional valence of words affects early stages of EP. This influence seems to be contingent upon factors such as life experience, motivational drives, and personality traits (Citron, 2012). Atypical modulation of word processing has been informed in patients with bipolar disorder (Ibáñez et al., 2014), chronic pain, anxiety, and on psychopaths (Kissler et al., 2006). In war contexts, words may be less relevant than visual stimuli. It is less likely to encounter survival-related information conveyed by words than by visual stimuli. This could explain why relative to Controls, Ex-combatants showed an atypical word valence processing (i.e., did not discriminate between word valences). This finding could not be due to lower literacy in Ex-combatants as both groups were matched according to their education. Future studies should further investigate the superiority of visual information over verbal information in Ex-combatants. For instance, whether emotions portrayed by scenes of real life events would have an impact on SCB variables similar to that described here for faces. It is necessary to identify the most efficient carriers of emotional valences in this population as this would create an opportunity to enhance communication and social skills via intervention programs.

One final and novel finding of this study is the informative association between Neutral errors during the Face task and the temporal dynamic of word processing (i.e., N170 Latency) with social assertion skills. The “auto-expression in social situations” and the “expression of anger and displeasure” were significantly predicted by behavioral and electrophysiological variables, respectively, drawn from the ERT. Interpreting responses to neutral stimuli have not been the focus of the literature on EP. However, the influence of neutral stimuli on EP has more recently become a topic of interest (Güntekin and Başar, 2014; Camfield et al., 2016; Da Silva et al., 2016; Trujillo et al., 2017). While some attribute a passive role to neutral stimuli as the baseline condition in Emotion Recognition Tasks (Sprengelmeyer et al., 1998; Kesler-West et al., 2001; Pessoa et al., 2002; Kilts et al., 2003), others suggest that correct neutral categorization is contingent upon a meticulous reading of embedded contextual cues (Anderson et al., 2003; Hugenberg and Bodenhausen, 2004). In our study, we found that Controls were more prompted to misclassify Neutral Faces attributing a different valence (i.e., Angry). This suggests that their EP system is reactive to the ambiguity generated by multi-valence contexts such as that created by the ERT (see Trujillo et al., 2017). However, Ex-combatants did not show such reactivity, suggesting that their ability to resolve emotional ambiguities may have been modified by war experiences. The fact that Ex-combatants presented with emotional undifferentiated mechanisms which tend to prioritize visual (Faces) over verbal (Word) stimuli, indicates that their cognitive architecture in general and specifically the one supporting EP has been reorganized to operate in an adaptive way which best meets the demands of aggressive environments.

We acknowledge a number of limitations of the current study. For instance, we did not use a formal psychiatric interview, drug screening tests, or self-report clinical questionnaires to gather the individual’s health history. Although, we collected personal information about these antecedents during the general interview, future studies should incorporate standardized assessment procedures to collect this information. This would be relevant to investigate whether the adaptive mechanisms described here characterize all Colombian Ex-combatants. It might be argued that the findings presented here might not necessarily reflect the influence of war experiences. However, we made every effort to ensure that our investigated groups could only be distinguished based on war experiences and not on any socio-demographic background measures. Nevertheless, we acknowledge that populations embedded in conflict zones are very heterogeneous and there may be a number of confounding variables which could modulate the effects reported here. Despite this limitation, we feel confident to suggest that our results do reflect the influence of war experiences. For instance, recent studies involving actors from the same conflict zone investigated here have reported very similar findings and have suggested that chronic exposure to war conflict can reshape the functional architecture of cognition (Trujillo et al., 2017; see also Quintero-Zea et al., 2017). Interestingly, Trujillo et al. (2017) suggested that such changes can be reverted via valid intervention approaches. Finally, we acknowledge that some effects might be underestimated due to sample size. Thus, the replication of the findings reported here in a larger population would be an important future step.

## Author Contributions

Supervision of the study: NT, ST, DP; conception and design the study: NT, ST, MP, AI, DP; draft the manuscript ST, NT, SV, JU, MP; data collection: ST, NT, MR, JR, SV; data analysis: JL, NT, ST, MP; discussion and session analysis: ST, NT, MP, SV, JR, MR, JL, JU, AI. All the authors agreed on the final version of this manuscript.

## Conflict of Interest Statement

The authors declare that the research was conducted in the absence of any commercial or financial relationships that could be construed as a potential conflict of interest.

## Footnotes

1 http://www.reintegracion.gov.co/en
