# Supplementary material for: Atypical Modulations of N170 Component during Emotional Processing and Their Links to Social Behaviors in Ex-combatants
Source: Front Hum Neurosci. 2017 May 23;11:244. doi: 10.3389/fnhum.2017.00244 (PMC5440593; doi:10.3389/fnhum.2017.00244)
Supplement: Supplementary file 1 [file Presentation_1.PDF]

Supplementary material-integral

Atypical modulations on emotional processing and their impact on social behaviors in ex-combatants

Supplementary material 1

This material present the pilot study performed in healthy university students in order to adapt the task for it use on the population.

Pilot Study

We adapt an Emotional recognition task using face and word stimuli (Ibanez et al, 2011). Our goal were to evaluate in a set a healthy adults its accuracy, variation on the reaction times across the task and electrophysiological modulation during faces and word processing (Stimulus Type Effect-STE). Our hypothesis is that accuracy on the emotional and neutral conditions will be overall larger than 80%. We also assumed that variation on the reaction time during emotional recognition task is an indicator of emotional valence processing. We expect that reaction time variation will reach significant effect for face conditions. Finally, we explore the presences of stimulus type effect on the EEG signal.

Methods

Participants

Fifteen volunteers with mean age of 22 (SD = 3) and average education of 16 (SD = 3) entered this pilot study in order to validate the valence of images and words. Participants were students enrolled in University Courses who took part in the study on volunteer basis. None

of them reported having had psychiatric or neurological problems, or symptoms of anxiety. They all signed a consent form prior participations.

Task

Emotional recognition task

A task for identifying faces and words with emotional content was designed in E-prime (Psychology Software Tools, Pittsburg, USA). Faces images were taken from the MMI Facial Expression Database (Pantic et al., 2005). This database control perceptual condition of the images, such as luminosity, intensity and face position (eg. Frontal, lateral). For the MMI Database student and staff from Delft University were instructed for an expert on FACS Coder to express different basic emotion and neutral expression on the face. After the collection of the dataset, two expert in FACS coder selected the images that best capture the emotional and neutral expressions. In addition, the pictures were presented to two observed in order to select the emotion or neutral expression that the faces were showing. If controversy appears the final decision of the inclusion of the image were taken by a third FACS expert (for details see Pantic et al., 2005).

From MMI Facial Expression Database we selected 120 images expressing happy, neutral or angry expression in frontal position. We presented the images to two university observers who were instructed to classify them as happy, angry or neutral. If both observers correctly categorize the image its was included. If controversies appear a member of the research group took the final decision. The final set includes 90 pictures of female and male faces (30 happy, 30 neutral and 30 angry). All the images were cropped oval shaped and

standardized under the same parameters of color, brightness and size according to the criteria used previously reported (Hurtado et al., 2009, Ibanez et al., 2011). In order to avoid strange cues such as hair and ears, all face images.

Words were selected from a linguistic corpus created by the Faculty of Communication of Universidad de Antioquia [http://comunicaciones.udea.edu.co/corpuslinguistico/?opcion=4] which contain the most frequent words use in Antioquia´s region, Colombia. This corpus is part of a large Spanish project in order to establish different characteristics of the production of the idiom around different Spanish spoken countries (PRESEEA, 2005). In Medellin, a 119 interviews was performed in a sample with difference socio-economic condition (González and Grajales 2012). Using the expertise of the linguistic group from communication faculty at University of Antioquia, we select 90 words (30 pleasant, 30 neutral and 30 unpleasant) whit high frequency expression across Medellin´s population. Stimuli were displayed in a 17 inches screen. The stimuli were presented 23 inches away from the participant´s eye field. Both faces and words were adapted following Ibanez et al. (2010), Petroni et al. (2011), Ibanez et al. (2011), and Hurtado et al. (2009). Faces and words shown in screen were controlled in brightness, color and intensity. They were presented in a 17 inches screen, 60 cm apart from the participant.

2.3.2 Emotion processing assessment

The task compressed 360 trials. Each trial started with a 1000-miliseconds fixation cross, followed by a 200-miliseconds stimulus (face or word), and finally a 700-1000 milliseconds inter-stimulus interval (ISI) (black screen) was displayed. For error-trials, a 100-miliseconds red cross was displayed between the response notice and the ISI in order to provide a negative

feedback and to encourage attention to the task. No feedback was provided in correct-trials. We measured accuracy and response time. The task was sync to collect electrophysiological signal associated with task performance.

Data recording

Behavioral and electrophysiological data recording and signal processing follows the same methodology presented in the paper.

Data analysis

A repeat measure ANOVA was used to evaluate accuracy and reaction time for faces and words. Post hoc analysis per condition was carry out using paired t test.

Repeat measure ANOVA was also used for the analysis of electrophysiological stimulus type on this sample. This model has three level integrated for Category (faces and words) per condition (e.g. face: Happy, neutral, angry, words: pleasant, unpleasant, neutral) per hemisphere (eg. right and left).

Results

Accuracy: There were difference on face conditions [$F(2, 13)=32$, $P= 0.00$; $\eta^2 = 0.83$, $\beta=1$]. Happy faces describe a larger accurate performance in comparison with neutral and angry faces [$t= 5.94$, $P=0.00$; $t= 10.11$, $P=0.00$]. No differences were found for the other combination [t= 1.32, p= 0.21]. A statistical tendency was identify for words [$F(2,13)=3,1$, $P= 0.06$; $\eta^2 = 0.42$, $\beta=0.55$].

Reaction time: Statistical difference were identified for reaction time performance during face condition [$F(2,13)=42,52$, $P= 0.00$; $\eta^2 = 0.87$, $\beta=1$]. Specifically, happy faces reach faster responses than neutral and angry respectively [$t= 6,9$, $P= 0.00$; t= 7,3, p= 0.00], whereas, no difference were observed for the comparison between neutral and angry stimulus [$t= 0.36$, $P= 0.72$]. No difference were observed on the reaction time of the word conditions [$F(2,13)=0.59$, $P= 0.51$; $\eta^2 = 0.31$, $\beta=0.15$]

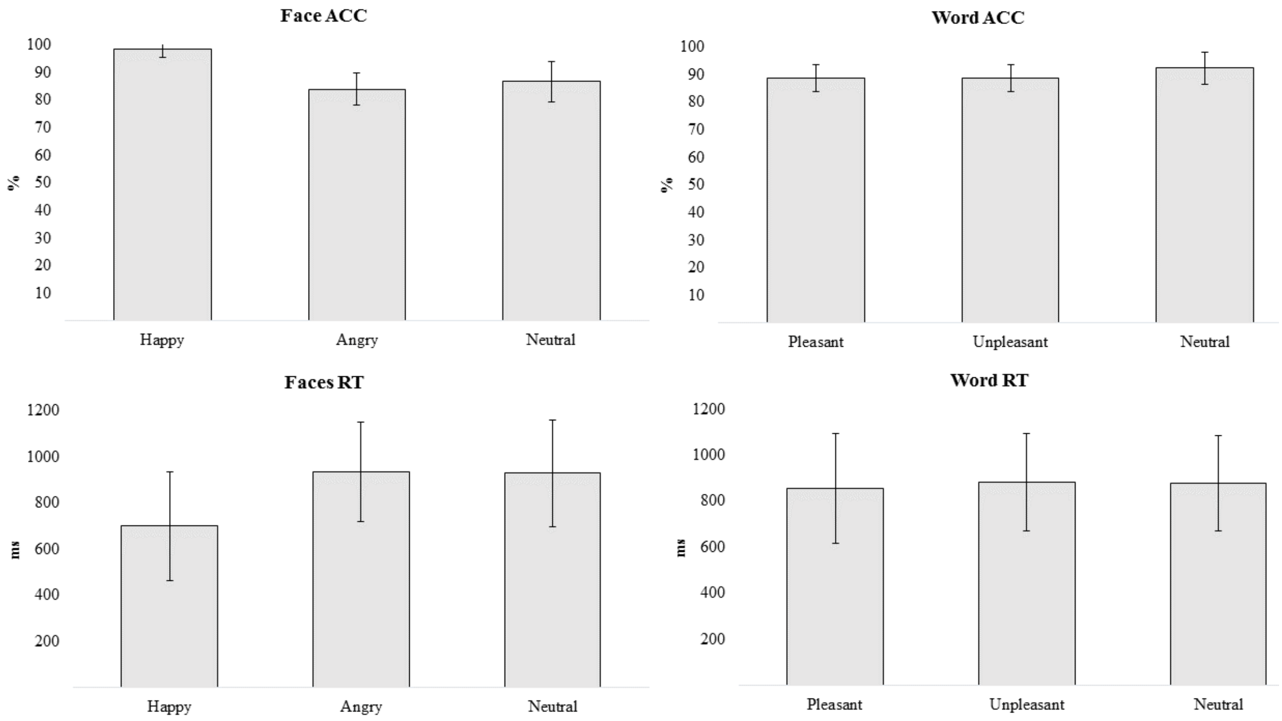

Supplementary Fig.1. Boxes and bars indicate of means and estandar deviation on face and word ccuracy (ACC) and reaction time (RT) condition in the Emotional Recognition Task (ERT).

Stimulus type effect

Greater neural activation was found for the faces in comparison with words conditions [F(1,14)= 13,49, P=0.00, η^2 =0.7, β=0.93]. This effect between categories describe a differential expression across hemispheres [F(1,14)= 36.47, P= 0,00 η^2 = 0.85, β=1]. Specifically, Post hoc test indicated that faces recruit more neural resources for the right than for the left hemisphere (t= 5.18, P=0.00). There were no differential use of neural resources across hemisphere for words (t=0.94, P=0.37).

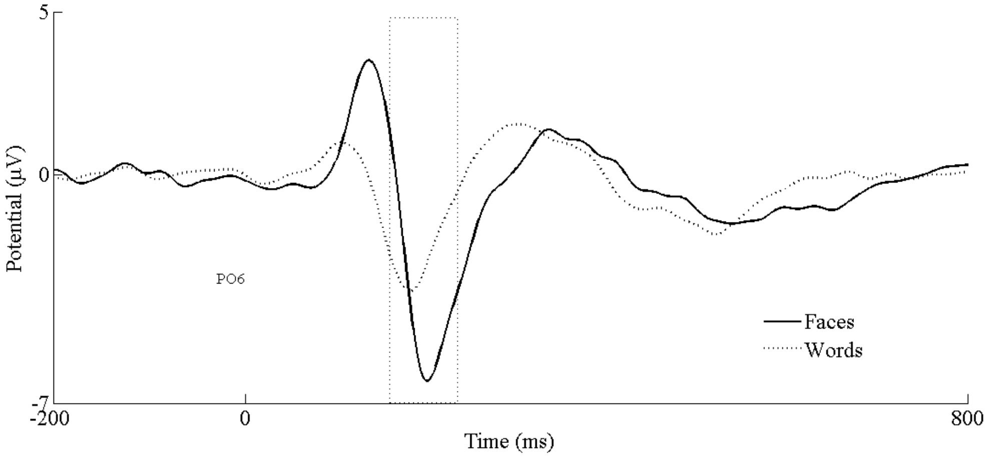

Supplementary figure 2. Stimulus type condition for word and face stimuli in healthy population.

Comments

The main goal of the pilot study was to identify behavioral performance in the task, in order to determine the utility of the task during emotional recognition discrimination, due to the analysis of accuracy, reaction time and stimulus type effect. On that sense, as we expected the average accuracy were overall close to 90%. In previous studies using emotional recognition task average accuracy was 80% or higher (Citron et al, 2012; Ibanez, et al 2011). That finding suggest that images selected were overall categorized with precision for

subjects in the sample. Complementary, accuracy modulation for face condition describe variability similar that previously reported by Sawada et al., (2014). Similar tendencies in accuracy for word conditions has been report previously (Citron et al., 2013)

Reaction time has been describe as a reflex of valence modulation. In our results, difference observed was for happy faces. This faster response has been interpreted as a facilitation for faces with positive content (Leppänen et al., 2003). For word, reaction time variation is overall less stable (Ibanez et al, 2011).

There was found Stimulus Type effect differences between word and face conditions. Specifically, variation on this has been describe previously as a marker distribution of differential neural resources for word and faces in emotional processing task (Rossion et al, 2003). Our data found larger right hemisphere distribution for face than for word conditions. In summary, this pilot study identify an overall accurate response, modulation of some of the core conditions of the reaction time associated with emotional valence and the electrophysiological Stimulus Type Effect modulation, indicate that in general term the task has replicate the core effect previous associated with other adaptations.

References

Citron, F. M. (2012). Neural correlates of written emotion word processing: a review of recent electrophysiological and hemodynamic neuroimaging studies. *Brain and Language*, *122*(3), 211-226.

Citron, F. M., Weekes, B. S., & Ferstl, E. C. (2013). Effects of valence and arousal on written word recognition: Time course and ERP correlates. *Neuroscience Letters*, *533*, 90-95.

González, M. C., & Grajales, R. (2012). La clase social en el Corpus Preseea-Medellín.

Hurtado, E., Haye, A., González, R., Manes, F., & Ibáñez, A. (2009). Contextual blending of ingroup/outgroup face stimuli and word valence: LPP modulation and convergence of measures. *BMC Neuroscience*, *10*(1), 69.

Ibanez, A., Gleichgerrcht, E., Hurtado, E., González, R., Haye, A., & Manes, F. F. (2010). Early neural markers of implicit attitudes: N170 modulated by intergroup and evaluative contexts in IAT. *Frontiers in Human Neuroscience*, *4*, 188.

Ibanez, A., Hurtado, E., Riveros, R., Urquina, H., Cardona, J. F., Petroni, A., Lobos-Infante, A., Barutta, J., Baez, S., & Manes, F. (2011). Facial and semantic emotional interference: a pilot study on the behavioral and cortical responses to the Dual Valence Association Task. *Behavioral and Brain Functions*, *7*(8).

Leppänen, J. M., Tenhunen, M., & Hietanen, J. K. (2003). Faster choice-reaction times to positive than to negative facial expressions: The role of cognitive and motor processes. *Journal of Psychophysiology*, *17*(3), 113.

Pantic, M., Valstar, M., Rademaker, R., & Maat, L. (2005). Web-based database for facial expression analysis. In *2005 IEEE international conference on multimedia and Expo* (pp. 317-321). IEEE. (www.mmifacedb.com)

Petroni, A., Canales-Johnson, A., Urquina, H., Guex, R., Hurtado, E., Blenkmann, A., von Ellenrieder N., Manes F., Sigman, M., & Ibanez, A. (2011). The cortical processing of facial emotional expression is associated with social cognition skills and executive functioning: a preliminary study. *Neuroscience Letters*, *505*(1), 41-46.

PRESEEA (2005). Estudios sociolingüísticos de Medellín. Fase. 1. Corpus sociolingüístico de Medellín. From http://comunicaciones.udea.edu.co/corpuslinguistico/web/corpus.pdf

Rossion, B., Joyce, C.A., Cottrell, G.W., Tarr, M.J. (2003). Early lateralization and orientation tuning for face, word, and object processing in the visual cortex. *Neuroimage*, 20(3), 1609–24.

Sawada, R., Sato, W., Uono, S., Kochiyama, T., & Toichi, M. (2014). Electrophysiological correlates of the efficient detection of emotional facial expressions. *Brain Research*, *1560*, 60-72.

Supplementary material 2

This material provides the main effect results for the two way ANOVA model. Condition by Group interactions are reported on the main manuscript.

Results

Experimental task: Behavioral analysis

Regarding accuracy, a main effect for condition was found for faces [F(2,45)=26.4, P=0.00, η^2=0.6, β=1]. Happy faces reach accurate responses than angry and neutrals (t=6,6, P=0.00)(t=7,2, P=0.00). No differences was found for neutral vs. angry (t=1,3, P=0.19). Differences were also found for the accuracy in words [F(2,45)=15,1, P=0.00, η^2=0.57, β= 0.99]. Where pleasant word describe larger performance differences in comparison with neutral and unpleasant (t=4,7, P=0.00),(t=2,1, P=0.05) whereas, unpleasant describe larger accuracy than neutrals (t=3,4, P=0.01)]. No main effect was informed for group [face F(2,45)=1.36, P=0.25, η^2=0.17, β=0.21; words F(2,45)=5.16, P=0.03, η^2=0.32, β=0.60]

For reaction time reach significance just for words but not for faces [F(2,45)=2.79, P=0.07, η^2=0.33, β=0.52; Words: F(2,45)=4.14, P=0.02, η^2=0.39, β= 0.70]. For the reaction time pleasant word describe shorter reaction time than neutral and unpleasant (t=2,0 P=0.05)(t=2,7 P=0.01), no presented for the neutral and unpleasant conditions (t=0,1 P=0.89). Group main effect yielded no difference face [F(2,45)=7.34, P=0.01, η^2=0.37, β=0,76] for words [F(2,45)=3.89, P=0.05, η^2=0.28, β=0,49].

For the Error Type on face condition, difference were found for the main effect on neutral Error Type [$F(1,47)=4.37$, $P=0.04$, $\eta^2=0.29$, $\beta=0.54$] (neutral error angry > neutral error happy) (MSE= 4.75, P= 0.04, IC= 0.18 to 9.33) nor for the main effect in group [$F(1,47)=0.18$, $P=0.67$, $\eta^2=0.06$, $\beta=0.07$]. For angry condition main effect on the Error Type were found [$F(1,47)=16.93$, $P=0.00$, $\eta^2=0.51$, $\beta=0.98$] (angry error neutral > angry error happy) (MSE= 12.80, P= 0.00, IC= 6.49 to 19.11) and for group [$F(1,47)=5.10$, $P=0.03$, $\eta^2=0.31$, $\beta=0.60$] (ex-combatants >controls) (MSE= 6.38, P= 0.03, IC= 0.70 to 12.06). No difference were found for happy [Error Type $F(1,47)=0.35$, $P=0.56$, $\eta^2=0.08$, $\beta=0.09$; Group $F(1,47)=0.97$, $P=0.33$, $\eta^2=0.14$, $\beta= 0.16$].

For word condition, main effect on the error type of pleasant condition was identity [$F(1,47)=5.84$, $P=0.02$, $\eta^2=0.32$, $\beta=0.63$] (pleasant error neutral > pleasant error unpleasant) (MSE= 5.29, P= 0.02, IC= 0.75 to 9.83) and group [$F(1,47)=5.70$, $P=0.02$, $\eta^2=0.32$, $\beta=0.65$] (ex-combatants >controls) (MSE= 6.23, P= 0.02, IC= 0.98 to 11.48) was identified. Neutral word inform main effect on the error type [$F(1,47)=76.14$, $P=0.00$, $\eta^2=0.79$, $\beta=1$] (neutral error pleasant > neutral error unpleasant) (MSE= 20.06, P= 0.00, IC= 15.43 to 24.68) no for group [$F(1,47)=1.99$, $P=0.17$, $\eta^2=0.20$, $\beta=0.28$]. For unpleasant no difference were found for the main effects of Error Type nor Group [$F(1,47)=0.27$, $P=0.60$, $\eta^2=0.08$, $\beta=0.08$; $F(1,47)=3.83$, $P=0.06$, $\eta^2=0.27$, $\beta=0.48$].
